# The 2017 ACC/AHA stage 1 hypertension is associated with arterial stiffness: a prospective analysis

**DOI:** 10.18632/aging.202764

**Published:** 2021-03-26

**Authors:** Shanshan Liu, Shujing Wu, Jingya Niu, Ruizhi Zheng, Lizhan Bie, Zhuojun Xin, Shuangyuan Wang, Hong Lin, Zhiyun Zhao, Mian Li, Tiange Wang, Min Xu, Jieli Lu, Yuhong Chen, Yiping Xu, Yufang Bi, Weiqing Wang, Guang Ning, Yu Xu

**Affiliations:** 1 Department of Endocrine and Metabolic Diseases, Shanghai Institute of Endocrine and Metabolic Diseases, Ruijin Hospital, Shanghai Jiaotong University School of Medicine, Shanghai, China; 2 Shanghai National Clinical Research Center for Metabolic Diseases, Key Laboratory for Endocrine and Metabolic Diseases of the National Health Commission of the PR China, Shanghai National Center for Translational Medicine, Ruijin Hospital, Shanghai Jiaotong University School of Medicine, Shanghai, China; 3 Clinical Trials Center, Ruijin Hospital, Shanghai Jiaotong University School of Medicine, Shanghai, China

**Keywords:** 2017 ACC/AHA guideline, stage 1 hypertension, arterial stiffness, cohort

## Abstract

Objective: To examine the association between stage 1 hypertension defined by the 2017 American College of Cardiology and American Heart Association (ACC/AHA) guideline and risk of developing arterial stiffness.

Methods: During 2010-2015, 4595 adults aged ≥40 years without cardiovascular disease were followed up for a median of 4.3 years. BP levels at baseline were categorized into normal, elevated, stage 1 hypertension, and stage 2 hypertension. The development of arterial stiffness was defined as a normal brachial-ankle pulse wave velocity (ba-PWV) at baseline and an increased ba-PWV at follow-up.

Results: Compared with participants with normal BP, participants with stage 1 hypertension had a 1.48-fold increased risk of developing arterial stiffness [odds ratio (OR) =2.48; 95% confidence interval (CI) =1.59-3.85] after adjustment for cardiovascular risk factors. The association was more evident in adults aged 40-59 years (OR =4.08; 95% CI =2.06-8.08) than that in those aged ≥60 years (OR =1.47; 95% CI =0.81-2.67). A systolic BP 130~139 mmHg was significantly associated with arterial stiffness independent of diastolic BP (OR =2.90; 95% CI =1.86-4.52). Stage 1 hypertension either at baseline or at follow-up was associated with increased risks compared with normal BP at both baseline and follow-up.

Conclusions: The 2017 ACC/AHA stage 1 hypertension was significantly associated with higher risks of arterial stiffness.

## INTRODUCTION

High blood pressure (BP) contributes to atherosclerosis and increases the risk of cardiovascular disease (CVD). In 2017, the American College of Cardiology and the American Heart Association (ACC/AHA) redefined hypertension as BP levels ≥130/80 mmHg and BP levels 130~139/80~89 mmHg which were regarded as ‘high-normal’ previously, were classified as stage 1 hypertension [1]. However, this definition was not embraced by other guidelines, such as the 2018 European Society of Cardiology/European Society of Hypertension (ESC/ESH) guidelines and the 2020 International Society of Hypertension (ISH) Global Hypertension Practice Guidelines [2–4]. Therefore, whether BP levels 130~139/80~89 mmHg should be defined as hypertension warrants investigation. Subsequent studies have reported significant associations between ACC/AHA stage 1 hypertension and risks of CVD events in long-term population-based cohorts [5–7], providing strong evidence and justification for the definition of ACC/AHA stage 1 hypertension in these populations for an early intervention.

Pulse wave velocity (PWV) was proposed as a method to assess arterial stiffness in 2003 [8] and has been widely accepted in cardiovascular studies [9]. Arterial stiffness defined by an increased PWV was considered a prelude to clinical atherosclerosis and was related to higher CVD risks. An individual participant meta-analysis revealed that PWV could predict future CVD events independent of conventional risk factors [10]. Brachial-ankle PWV (ba-PWV) is measured by brachial and tibial arterial wave analyses and previous studies have demonstrated significantly higher risks of CVD occurrence in association with increased ba-PWV levels independent of traditional cardiovascular risk factors in different populations [11–14]. Therefore, early detection and prevention of arterial stiffness are of great importance.

Previous studies have shown significant associations between BP levels and arterial stiffness. For instance, subjects with a high level of ba-PWV had higher risks of hypertension [15], and subjects with a high BP level were more likely to have increased ba-PWV [16]. However, most studies were cross-sectional. Associations of stage 1 hypertension defined by the 2017 ACC/AHA guidelines with the development of arterial stiffness were rarely reported. Therefore, using data from a well-defined population cohort of community residents in China, we aimed to examine the associations between ACC/AHA stage 1 hypertension and incident arterial stiffness defined by an increased ba-PWV.

## RESULTS

General characteristics of study participants are described in Table 1. Among 4595 participants, 699 (15.2%) had normal BP, 528 (11.5%) had elevated BP, 1099 (23.9%) had stage 1 hypertension, and 2269 (49.4%) had stage 2 hypertension including 1,008 participants using anti-hypertensive medications. Compared with participants in the normal BP category, participants in the higher BP categories were older and more likely to be men, had higher levels of body mass index (BMI), fasting plasma glucose (FPG), triglycerides, total cholesterol, low-density lipoprotein (LDL) cholesterol, and urinary albumin-to-creatine ratio (ACR), and had lower levels of education, high-density lipoprotein (HDL) cholesterol, and estimated glomerular filtration rate (eGFR) (all P for trend <0.001). In addition, baseline ba-PWV levels increased significantly across BP categories (P for trend <0.001).

**Table 1:** Baseline characteristics according to blood pressure categories.

**Characteristics**	**Total n=4595**	**Blood pressure categories**				***P _for trend_***
		**Normal BP n=699**	**Elevated BP n=528**	**Stage 1 hypertension n=1099**	**Stage 2 hypertension n=2269**	
Systolic blood pressure (mmHg)	136.0 ± 17.0	112.1 ± 5.6	124.6 ± 2.9 **	130.8 ± 6.4 **	148.6 ± 13.4 **	<0.001
Diastolic blood pressure (mmHg)	82.0 ± 9.8	70.8 ± 5.2	73.6 ± 4.8 **	81.4 ± 5.3 **	87.6 ± 8.9 **	<0.001
Age (years)	55.8 ± 7.7	53.0 ± 7.2	56.2 ± 7.8 **	54.4 ± 7.4 **	57.2 ± 7.6 **	<0.001
Men, n (%)	1684 (36.65)	196 (28.04)	167 (31.63)	425 (38.67) **	896 (39.49) **	<0.001
Education ≥9 years, n (%)	3256 (71.17)	556 (79.77)	372 (70.59) **	805 (73.45) **	1523 (67.54) **	<0.001
Current smoking, n (%)	972 (21.92)	137 (20.24)	107 (21.19)	275 (25.89) **	453 (20.68)	0.980
Current drinking, n (%)	464 (10.44)	52 (7.64)	35 (6.97)	127 (11.94) **	250 (11.38) **	0.001
Physical activity ≥600 METs-min/week, n (%)	3305 (71.93)	511 (73.10)	383 (72.54)	780 (70.97)	1631 (71.88)	0.537
Body mass index (kg/m^2^)	25.0 ± 3.2	23.2 ± 2.7	24.2 ± 2.7 **	24.7 ± 2.9 **	26.0 ±3.3 **	<0.001
Fasting plasma glucose (mg/dL)	92.07 (85.23-101.08)	87.93 (81.98-94.23)	90.45 (84.32-97.48) **	91.89 (85.05-100.18) **	94.23 (87.03-104.32) **	<0.001
Triglycerides (mg/dL)	100.00 (71.42-140.60)	81.20 (60.90-113.53)	87.07 (63.91-127.44) **	99.25 (72.93-139.10) **	109.02 (78.20-154.14) **	<0.001
Low-density lipoprotein cholesterol (mg/dL)	122.52 ± 32.27	120.73 ± 31.10	120.73 ± 31.10	121.73 ± 33.02 **	124.95 ± 32.08 **	<0.001
High-density lipoprotein cholesterol (mg/dL)	51.44 ± 12.38	53.46 ± 12.31	52.65 ± 12.79	52.07 ± 13.05 *	50.23 ± 11.84 **	<0.001
Total cholesterol (mg/dL)	204.99 ± 37.08	197.59 ± 36.03	201.85 ± 36.20 *	205.40 ± 37.66 **	207.80 ± 36.98 **	<0.001
eGFR (mL/min/1.73m^2^)	101.75 (95.68-107.29)	104.07 (97.97-109.30)	101.99 (95.95-107.41) **	103.19 (96.79-108.30) **	100.22 (94.48-105.98) **	<0.001
Urinary albumin-to-creatine ratio (mg/g)	4.52 (2.64-8.05)	3.90 (2.59-6.45)	4.10 (2.36-6.63)	4.44 (2.62-7.67) **	4.91 (2.75-9.26) **	<0.001
Ba-PWV (cm/s)	1446 (1310-1593)	1273 (1175-1375)	1365 (1259-1503) **	1409 (1298-1545) **	1536 (1408-1649) **	<0.001

Continuous variables are presented as means ± standard deviation or medians (interquartile ranges). Categorical variables are presented as n (%). * *P* <0.05; ** *P* <0.01 *vs.* normal BP group.

METs-min/week, metabolic equivalents-minute per week; eGFR, estimated glomerular filtration rate; Ba-PWV, brachial-ankle pulse wave velocity.

**Figure 1:**
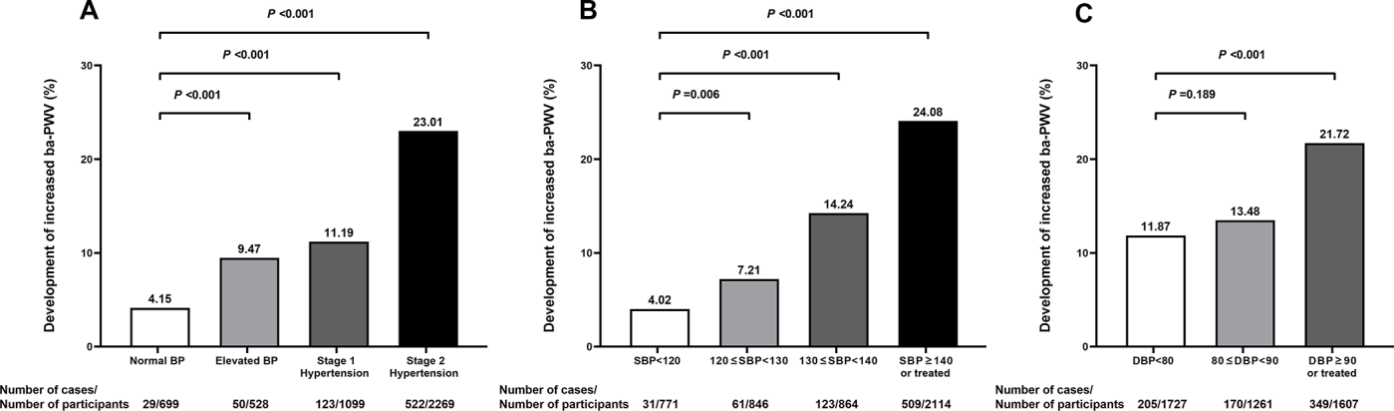
**Proportions (%) of participants developing an increased ba-PWV in different blood pressure categories.** 2017 ACC/AHA blood pressure categories (**A**); systolic blood pressure categories (**B**); diastolic blood pressure categories (**C**). SBP, systolic blood pressure; DBP, diastolic blood pressure; Ba-PWV, brachial-ankle pulse wave velocity.

After a median of 4.3 years’ follow-up, 724 participants developed an increased ba-PWV (Figure 1), which was defined as a normal ba-PWV at baseline (≤1793 cm/s, i.e. the 75th percentile of baseline ba-PWV) and an increased ba-PWV at follow-up (>1793 cm/s). Proportions of participants developing arterial stiffness were 4.15%, 9.47%, 11.19%, and 23.01% in groups of normal BP, elevated BP, stage 1 hypertension, and stage 2 hypertension, respectively. Significant differences were found for the development of arterial stiffness between participants with stage 1 hypertension and participants with normal BP, or between participants with systolic BP 130~139 mmHg and participants with systolic BP <120 mmHg (both P <0.001). However, no difference was found between participants with diastolic BP 80~89 mmHg and participants with diastolic BP <80 mmHg (P = 0.189).

Risks of developing arterial stiffness in association with BP categories are shown in Table 2. Compared with participants with normal BP, participants with stage 1 hypertension had a 1.48-fold increased risk of developing arterial stiffness [odds ratio (OR) =2.48; 95% confidence interval (CI) =1.59-3.85, P <0.001] after adjustment for age, sex, education, current smoking, current drinking, physical activity, BMI, FPG, triglycerides, LDL cholesterol, HDL cholesterol, eGFR, and urinary ACR. Similar and significant associations between stage 1 hypertension and risks of developing arterial stiffness were found in men and women, in participants with or without dysglycemia after full adjustment (both P for interaction >0.1). However, a significant interaction was observed for age (P for interaction =0.039). In participants aged <60 years, stage 1 hypertension was associated with a 3.08-fold increased risk of developing arterial stiffness (OR =4.08; 95% CI =2.06-8.08, P <0.001) whereas in participants aged ≥60 years, no significant association was found.

**Table 2:** Risks of developing an increased ba-PWV in association with blood pressure categories in the overall study population and in subgroups of sex, age, and dysglycemia.

**Blood pressure categories**	**Odds ratio (95% confidence interval)**											
	**Total**				**Sex †**			**Age †**			**Dysglycemia †**	
	**Model 1**	**Model 2**	**Model 3**		**Men**	**Women**		**<60 years**	**≥60 years**		**Without**	**With**
Normal BP	1.00	1.00	1.00		1.00	1.00		1.00	1.00		1.00	1.00
Elevated BP	**1.73 (1.06-2.82)**	**1.65 (1.00-2.73)**	1.59 (0.96-2.63)		1.20 (0.53-2.72)	1.90 (0.99-3.65)		**2.94 (1.34-6.43)**	1.07 (0.55-2.08)		1.80 (0.95-3.42)	1.26 (0.55-2.91)
Stage 1 hypertension	**2.68 (1.75-4.11)**	**2.70 (1.75-4.19)**	**2.48 (1.59-3.85)**		**2.14 (1.09-4.24)**	**2.74 (1.52-4.93)**		**4.08 (2.06-8.08)**	1.47 (0.81-2.67)		**2.14 (1.20-3.82)**	**2.64 (1.29-5.41)**
Stage 2 hypertension	**5.05 (3.40-7.51)**	**5.10 (3.38-7.68)**	**4.54 (3.00-6.87)**		**3.64 (1.92-6.90)**	**5.34 (3.08-9.24)**		**8.04 (4.17-15.52)**	**3.01 (1.75-5.20)**		**5.14 (3.03-8.71)**	**3.90 (1.97-7.71)**
*P _for trend_*	<0.001	<0.001	<0.001		<0.001	<0.001		<0.001	<0.001		<0.001	<0.001
*P _for interaction_*					0.260			0.039			0.609	

Model 1: adjusted for age, sex.

Model 2: further adjusted for education, current smoking, current drinking, physical activity, and body-mass index.

Model 3: further adjusted for fasting plasma glucose, triglycerides, low-density lipoprotein cholesterol, high-density lipoprotein cholesterol, estimated glomerular filtration rate, urinary albumin-to-creatine ratio.

† Using model 3 except the stratified variable.

Ba-PWV, brachial-ankle pulse wave velocity.

Risks of developing arterial stiffness in association with systolic and diastolic BP categories separately are shown in Table 3. Compared with participants with a systolic BP <120 mmHg, participants with a systolic BP 130~139 mmHg had a 2.03-fold increased risk of developing arterial stiffness (OR =3.03; 95% CI =1.97-4.67, P <0.001) after adjustment for confounding factors. Further adjustment for diastolic BP did not alter the association (OR =2.90; 95% CI =1.86-4.52, P <0.001). Compared with participants with a diastolic BP <80 mmHg, participants with a diastolic BP 80~89 mmHg had a 50% increased risk of developing arterial stiffness (OR =1.50; 95% CI =1.18-1.91, P <0.001) after adjustment for confounding factors. However, further adjustment for systolic BP attenuated the association to the null (OR =1.09; 95% CI =0.85-1.41, P =0.492).

**Table 3:** Risks of developing an increased ba-PWV in association with systolic blood pressure or diastolic blood pressure categories separately.

**Blood pressure categories**	**Odds ratio (95% confidence interval)**			
	**Model 1**	**Model 2**	**Model 3**	**Model 4**
SBP (mmHg)				
<120	1.00	1.00	1.00	1.00
120~129	1.57 (0.99-2.47)	1.52 (0.95-2.43)	1.44 (0.90-2.30)	1.41 (0.88-2.26)
130~139	**3.26 (2.15-4.95)**	**3.28 (2.13-5.03)**	**3.03 (1.97-4.67)**	**2.90 (1.86-4.52)**
≥140 or treated	**5.25 (3.58-7.69)**	**5.33 (3.58-7.92)**	**4.74 (3.18-7.08)**	**4.43 (2.87-6.83)**
*P _for trend_*	<0.001	<0.001	<0.001	<0.001
DBP (mmHg)				
<80	1.00	1.00	1.00	1.00
80~89	**1.54 (1.22-1.95)**	**1.55 (1.22-1.96)**	**1.50 (1.18-1.91)**	1.09 (0.85-1.41)
≥90 or treated	**2.37 (1.93-2.90)**	**2.27 (1.83-2.83)**	**2.12 (1.70-2.66)**	1.22 (0.94-1.58)
*P _for trend_*	<0.001	<0.001	<0.001	0.130

Model 1: adjusted for age, sex.

Model 2: further adjusted for education, current smoking, current drinking, physical activity, and body-mass index.

Model 3: further adjusted for fasting plasma glucose, triglycerides, low-density lipoprotein cholesterol, high-density lipoprotein cholesterol, estimated glomerular filtration rate, urinary albumin-to-creatine ratio.

Model 4: further adjusted for DBP (in association with SBP categories) or SBP (in association with DBP categories).

Ba-PWV, brachial-ankle pulse wave velocity; SBP, systolic blood pressure; DBP, diastolic blood pressure.

**Figure 2:**
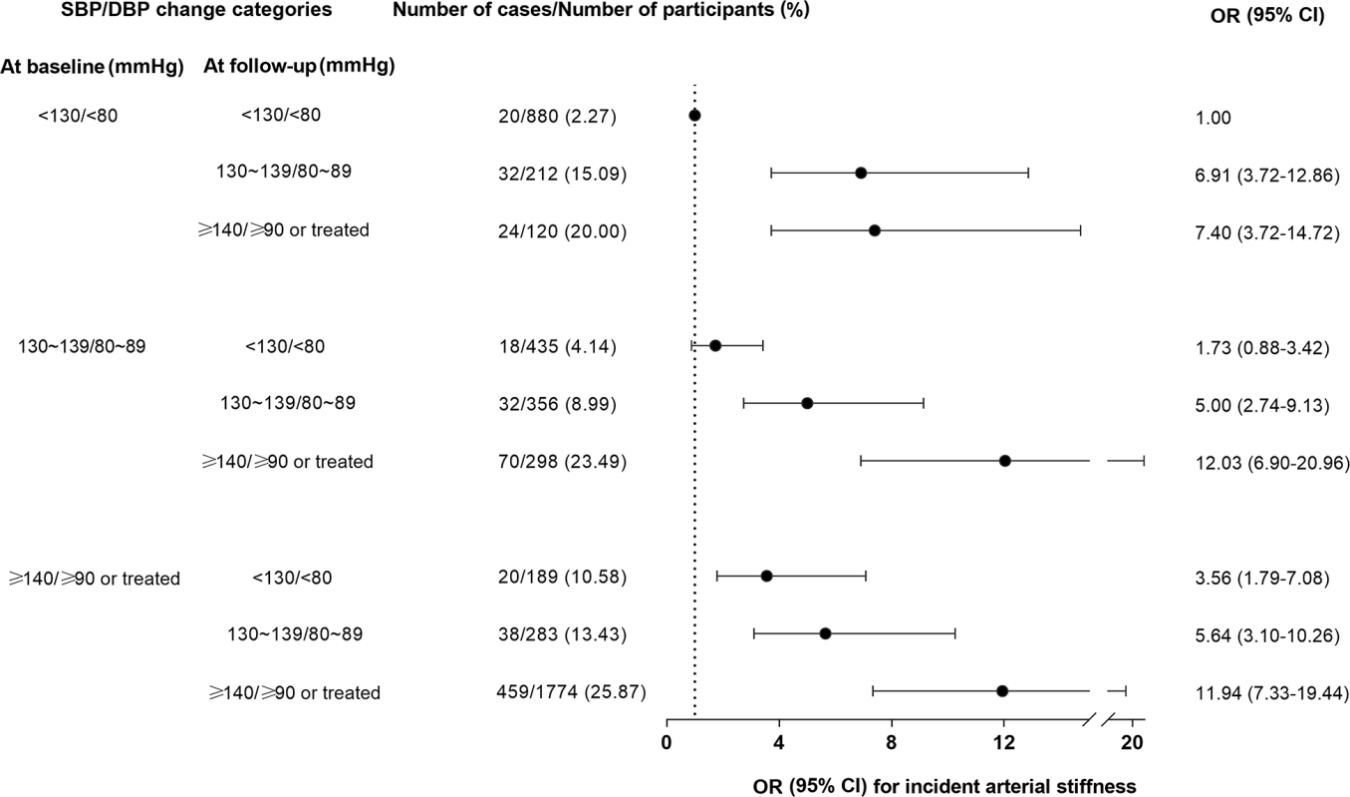
**Risks of developing an increased ba-PWV in association with blood pressure changes during follow-up.** The OR (95% CI) was adjusted for age, sex, education, current smoking, current drinking, physical activity, body-mass index, fasting plasma glucose, triglycerides, low-density lipoprotein cholesterol, high-density lipoprotein cholesterol, estimated glomerular filtration rate, and urinary albumin-to-creatine ratio. Forty-eight participants had missing data on BP measurements during follow-up and were not included in the analysis. BP, blood pressure; SBP, systolic blood pressure; DBP, diastolic blood pressure; Ba-PWV, brachial-ankle pulse wave velocity; OR, odds ratio; CI, confidence interval.

Further categorization using both baseline BP and follow-up BP and their associations with arterial stiffness are shown in Figure 2. Using participants with systolic BP <130 mmHg and diastolic BP <80 mmHg at both baseline and follow-up as the reference, participants whose BP levels were <130/<80 mmHg at baseline and 130~139/80~89 mmHg at follow-up had a 5.91-fold increased risk of arterial stiffness (OR =6.91; 95% CI =3.72-12.86, P<0.001). For participants with BP levels 130~139/80~89 mmHg at baseline, significantly increased risks of arterial stiffness were found for BP levels 130~139/80~89 mmHg or higher at follow-up.

## DISCUSSION

Using data from a well-defined community cohort of Chinese adults aged ≥40 years, we found that stage 1 hypertension defined by the 2017 ACC/AHA guideline was significantly associated with a higher risk of developing arterial stiffness defined by an increased ba-PWV level compared with the normal BP category. The association was more evident in adults aged 40-59 years and was not significant in adults aged ≥60 years. In addition, a systolic BP of 130~139 mmHg might be more closely related with arterial stiffness than a diastolic BP 80~89 mmHg. Furthermore, participants with BP levels 130~139/80~89 mmHg either at baseline or at follow-up had increased risks of arterial stiffness compared with continuously normal BP.

Although there have been studies reporting significant associations between the newly defined stage 1 hypertension by the ACC/AHA and major clinical cardiovascular events in different populations [5–7, 17–20], few studies have assessed the impact of stage 1 hypertension on arterial stiffness. One cross-sectional study examined the association of the 2017 ACC/AHA BP categorization with ba-PWV levels in 772 Afro-Caribbean men aged ≥50 years [21]. They found significantly higher ba-PWV levels in men with ACC/AHA stage 1 hypertension. Findings from the current study were consistent with previous findings and extended the evidence by examining prospectively the association between stage 1 hypertension and the development of arterial stiffness in a general Chinese population. One longitudinal study examined the association of systolic BP and the rates of change in PWV in 775 US adults [16]. They found that the effect of systolic BP on PWV trajectories exists in the prehypertensive range. However, prehypertension defined in this study was according to the JNC 7 guideline (i.e. systolic BP 120~139 mmHg). The new definition of stage 1 hypertension according to the 2017 ACC/AHA guideline (systolic/diastolic BP 130~139/80~89 mmHg) is the focus of the current study. According to our findings, participants with ACC/AHA stage 1 hypertension might already have an increased risk of arterial stiffness and therefore warrant an early intervention to prevent clinical cardiovascular events.

We found a significant interaction of age with the association between stage 1 hypertension and arterial stiffness. The significant association was observed in participants aged 40-59 years whereas in participants aged ≥60 years, the significant association was only found for stage 2 hypertension. The more evident association found in middle-aged adults compared with elderly adults was also demonstrated in previous studies for stage 1 hypertension and clinical cardiovascular outcomes. One study in 15 million Koreans aged 20-94 years found that stage 1 hypertension was associated with higher risks of CVD events compared with normal BP at all adult ages and the age group of 35-49 years had the highest relative risk [5]. Another study in 21,441 Chinese aged ≥35 years found significant associations between stage 1 hypertension and CVD events in participants aged 35-59 years, which was not found in participants aged ≥60 years [6]. Findings from the current study revealed that this age-specific association also exists for stage 1 hypertension and arterial stiffness. An appropriate high level of systolic BP might be required to maintain coronary perfusion in the elderly [22], leading to a potentially age-dependent threshold for hypertension [23]. Therefore, the definition of stage 1 hypertension in the elderly needs more evidence.

When systolic and diastolic BP were examined separately, we found systolic BP levels in accordance with stage 1 hypertension (130~139 mmHg) were significantly associated with arterial stiffness independent of diastolic BP whereas diastolic BP levels in accordance with stage 1 hypertension (80~89 mmHg) were no longer associated with arterial stiffness after adjustment for systolic BP, indicating a closer relation between systolic BP and arterial stiffness. Although stronger associations for systolic BP over diastolic BP in general for clinical cardiovascular outcomes have been demonstrated previously [24–26], a comparison between systolic BP and diastolic BP in stage 1 hypertension for its association with cardiovascular risks is limited [27]. A recent study in US adults found that both systolic and diastolic hypertension influenced the risk of adverse cardiovascular events, regardless of the definition of hypertension (≥140/90 mmHg or ≥130/80 mmHg). However, no further adjustment for systolic BP or diastolic BP was made for the association [28]. The stronger association of systolic BP 130~139 mmHg with arterial stiffness found in the current study provided further evidence for targeting systolic BP as the priority if the stage 1 hypertension is to be treated.

In addition, changes in BP levels over a median of 4.3 years were also important with regard to arterial stiffness, as found in the current study. Increasing BP levels during follow-up were all associated with increasing risks of arterial stiffness when compared with non-hypertensive BP levels maintained at both baseline and follow-up. Stage 1 hypertension, whether already present at baseline or developed during follow-up, was associated with a significantly increased risk of arterial stiffness, indicating the importance of long-term BP management under the levels of 130/80 mmHg in reducing cardiovascular risks.

The current study is among the first to examine prospectively the association between stage 1 hypertension and the development of arterial stiffness. Arterial stiffness was evaluated in a large sample of community residents using ba-PWV at baseline and at follow-up after a median of 4.3 years. BP levels of stage 1 hypertension were examined comprehensively by baseline systolic BP and diastolic BP combined or separated, as well as by changes in BP levels during follow-up. However, there are also limitations. First, BP levels were measured on a single day in the morning of clinic examination, although 3 measurements were obtained for each participant. Measuring BP levels at different times of the day and at different days is important for the diagnosis of hypertension. Second, the follow-up duration was relatively short and only one follow-up visit was conducted. Therefore, changes in BP levels and the development of arterial stiffness in the current study were recorded in parallel. Third, a significant number of participants with missing data on ba-PWV at follow-up were excluded, which has the potential to introduce selection bias. Although general characteristics were largely similar between participants included and those excluded, proportions of men and current smoking were higher and levels of BMI, BP, LDL cholesterol, and total cholesterol were lower in those excluded (Supplementary Table 1). Finally, although a comprehensive cardiometabolic risk profile was adjusted in multivariable models, residual or undetected confounders could not be ruled out.

In conclusion, the 2017 ACC/AHA stage 1 hypertension was significantly associated with higher risks of arterial stiffness evaluated by ba-PWV measurements. Systolic BP of stage 1 hypertension levels was associated with arterial stiffness independent of diastolic BP and should be prioritized for BP management if stage 1 hypertension is to be treated. In addition, long-term BP control under 130/80 mmHg might have to be maintained in order to prevent arterial stiffness. Future studies and more evidence are needed to determine whether treating stage 1 hypertension can reduce the risk of clinical and subclinical cardiovascular disease.

## MATERIALS AND METHODS

### Study population

**Figure 3:**
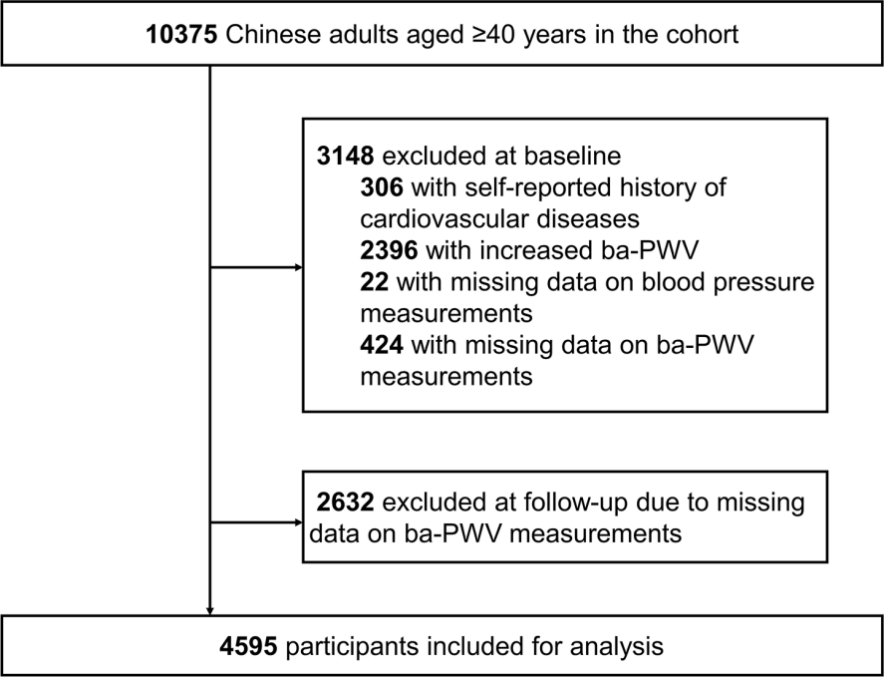
**Flow chart of the selection of study participants.** Ba-PWV, brachial-ankle pulse wave velocity.

The study was conducted among the residents living in Jiading District, Shanghai, China. The study design has been published elsewhere [29]. In 2010, 10375 registered permanent residents aged 40 years or older were recruited for a comprehensive examination of cardiometabolic health. During 2014 to 2015, participants were asked to come back for a follow-up visit. For the current analysis, we excluded 306 participants with self-reported history of CVD and 22 participants with missing data on BP measurements at baseline. We further excluded participants with missing data on ba-PWV at baseline or at follow-up and participants with already increased baseline ba-PWV levels. Eventually, 4595 participants were included for the current analysis. Details of the study population selection are presented in Figure 3.

The study protocol was approved by the Institutional Review Board at Ruijin Hospital affiliated to Shanghai Jiaotong University School of Medicine. All participants provided written informed consent.

### Data collection

At the baseline examination, each participant completed a standard questionnaire including sociodemographic characteristics, medical history of chronic diseases, and lifestyle factors, etc. Current smoking or current drinking was defined as smoking or drinking regularly during the past 6 months. Physical activity was recorded using the International Physical Activity Questionnaire (IPAQ) and was dichotomized according to the recommendation of current guidelines [≥600 metabolic equivalent minutes per week (MET-min/week)] [30]. Anthropometric measurements were performed according to a standard protocol. Height and weight were measured with participants wearing light clothes and without shoes. BMI was calculated as weight in kilograms divided by height in meters squared. Three BP measurements were obtained from each participant in a seated position using a calibrated automatic electronic device (OMRON Model HEM-752 FUZZY, Omron Company, Dalian, China) in a separate examination room after at least 5-minute sitting rest. Participants were advised to avoid alcohol, coffee, tea, smoking and exercise at least 30 minutes before BP measurement. The average of three readings was used for analysis.

Fasting venous blood sample was drawn from each participant after at least a 10-hour overnight fast. An oral glucose tolerance test was performed and blood samples were re-taken 2 hours after the glucose load. Plasma glucose, serum lipids and creatinine were measured on an auto-analyser (Modular P800, Modular E170; Roche, Basel, Switzerland). eGFR was calculated using the Chronic Kidney Disease Epidemiology Collaboration (CKD-EPI) equation [31]. A first voided urine sample at early morning was collected from each participant and urinary albumin and creatinine were measured to estimate the urinary ACR in mg/g.

### Ba-PWV measurement

Levels of ba-PWV were measured at the baseline and follow-up visits by a trained physician using Colin VP-1000 (Model BP203RPE II, form PWV/ABI; OMRON Colin Medical Instruments, Tokyo, Japan) [32]. After cuffs were placed on both sides of upper arms and ankles, pulse waves were obtained simultaneously. The time of delay and the distance from right and left upper arms to right and left ankles were used to calculate the right and left ba-PWV. The larger reading of the right and left ba-PWV was used for analysis. Because there is no clinical cutoff point for ba-PWV, we used the upper quartile of ba-PWV levels at baseline to define normal and increased ba-PWV, as described previously [33]. Therefore, the development of an increased arterial stiffness was defined as a normal ba-PWV (≤1793 cm/s) at baseline and an increased ba-PWV (>1793 cm/s) at follow-up.

### BP categories

BP was categorized according to the 2017 ACC/AHA guideline into four groups: 1) normal, systolic BP <120 mmHg and diastolic BP <80 mmHg; 2) elevated, systolic BP 120~129 mmHg and diastolic BP <80 mmHg; 3) stage 1 hypertension, systolic BP 130~139 mmHg and/or diastolic BP 80~89 mmHg; and 4) stage 2 hypertension, systolic BP ≥140 mmHg and/or diastolic BP ≥90 mmHg and/or using anti-hypertensive medications [1]. Furthermore, systolic BP and diastolic BP were categorized separately into systolic BP <120, 120~129, 130~139, and ≥140 mmHg or diastolic BP <80, 80~89, and ≥90 mmHg. Participants using anti-hypertensive medications were categorized in the highest BP group.

### Statistical analysis

Characteristics of study participants were described in total and by BP categories. Continuous variables are presented as means ± standard deviations (SDs) or medians (interquartile ranges) for skewed variables. Categorical variables are shown in absolute numbers (percentages). Linear regression analysis for continuous variables and chi-square tests for categorical variables were conducted to assess the significance of trend across BP categories. The skewed variables such as triglycerides and urinary ACR were logarithmically transformed before analysis. The percentages of participants who developed arterial stiffness at follow-up were calculated in each BP categories and were compared with the normal BP group using chi-square tests. The risks of developing arterial stiffness in association with BP categories were examined using logistic regression models with the normal BP group as the reference. The multivariable-adjusted OR (95% CI) were also calculated in subgroups of men or women, age <60 years or ≥60 years, with or without dysglycemia, and interactions were tested by adding a multiplicative term to the model. In addition, associations were examined with systolic BP and diastolic BP categories separately. Logistic regression models included model 1 with adjustment for age and sex, model 2 with further adjustment for education, current smoking, current drinking, physical activity, and BMI, and model 3 with additional adjustment for FPG, triglycerides, LDL cholesterol, HDL cholesterol, eGFR, and urinary ACR. Furthermore, associations between changes in BP levels over a 4.3-year follow-up period and risks of arterial stiffness were also examined, using logistic regression models adjusted for cardiovascular risk factors mentioned above. Participants were stratified by baseline and follow-up BP (<130/<80, 130~139/80~89, and ≥140/≥90 mmHg or treated) and cross-combined into 9 categories. Participants with systolic BP <130 mmHg and diastolic BP <80 mmHg at both baseline and follow-up were used as the reference.

A two-sided P value <0.05 was considered statistically significant. All analyses were performed with SAS software version 9.4 (SAS institute, Cary, NC).

## Supplementary Material

Supplementary Table 1
