# Supplementary material for: The 2017 ACC/AHA stage 1 hypertension is associated with arterial stiffness: a prospective analysis
Source: Aging (Albany NY). 2021 Mar 26;13(7):10075–86. doi: 10.18632/aging.202764 (PMC8064197; doi:10.18632/aging.202764)
Supplement: Supplementary Table 1 [file aging-13-202764-s001.pdf]

SUPPLEMENTARY TABLE

Supplementary Table 1. Baseline characteristics of participants included and those excluded due to missing follow-up ba-PWV.

Characteristics	Participants included in this study, *n*=4595	Participants excluded due to missing follow-up ba-PWV, *n*=2632	*P* value
Age (years)	55.8 ± 7.7	55.8 ± 9.3	0.910
Men, n (%)	1684 (36.65)	1081 (41.07)	<0.001
Education ≥9 years, n (%)	3256 (71.17)	3256 (71.17)	0.097
Current smoking, n (%)	972 (21.92)	627 (24.53)	0.012
Current drinking, n (%)	464 (10.44)	281 (11.00)	0.466
Physical activity ≥600 METs-min/week, n (%)	3305 (71.93)	1872 (71.12)	0.467
Body mass index (kg/m^2)	25.0 ± 3.2	24.8 ± 3.2	0.003
Systolic blood pressure (mmHg)	136.0 ± 17.0	134.4 ± 17.6	<0.001
Diastolic blood pressure (mmHg)	82.0 ± 9.8	81.4 ± 10.0	0.018
Fasting plasma glucose (mg/dL)	92.07 (85.23-101.08)	91.71 (85.23-99.82)	0.131
Triglycerides (mg/dL)	100.00 (71.42-140.60)	97.74 (69.92-140.60)	0.346
Low-density lipoprotein cholesterol (mg/dL)	122.52 ± 32.27	120.40 ± 32.55	0.007
High-density lipoprotein cholesterol (mg/dL)	51.44 ± 12.38	51.02 ± 12.16	0.159
Total cholesterol (mg/dL)	204.99 ± 37.08	202.06 ± 39.02	0.002
eGFR (mL/min/1.73m^2)	101.75 (95.68-107.29)	102.01 (94.70-107.76)	0.073
Urinary albumin-to-creatine ratio (mg/g)	4.52 (2.64-8.05)	4.48 (2.64-8.00)	0.590
Ba-PWV at baseline (cm/s)	1446 (1310-1593)	1435 (1296-1583)	0.075

Continuous variables are presented as means ± standard deviations or medians (interquartile ranges). Categorical variables are presented as n (%).
BP, blood pressure; METs-min/week, metabolic equivalents-minute per week; eGFR, estimated glomerular filtration rate; Ba-PWV, brachial-ankle pulse wave velocity.
